# Supplementary material for: Novel Driver Strength Index highlights important cancer genes in TCGA PanCanAtlas patients
Source: PeerJ. 2022 Aug 11;10:e13860. doi: 10.7717/peerj.13860 (PMC9375969; doi:10.7717/peerj.13860)
Supplement: Supplemental Information 2 [file peerj-10-13860-s002.zip › COHORTS/genes plots/detailed/2021_8_16_14_9_distribution_events_detailed_females_39.pdf]

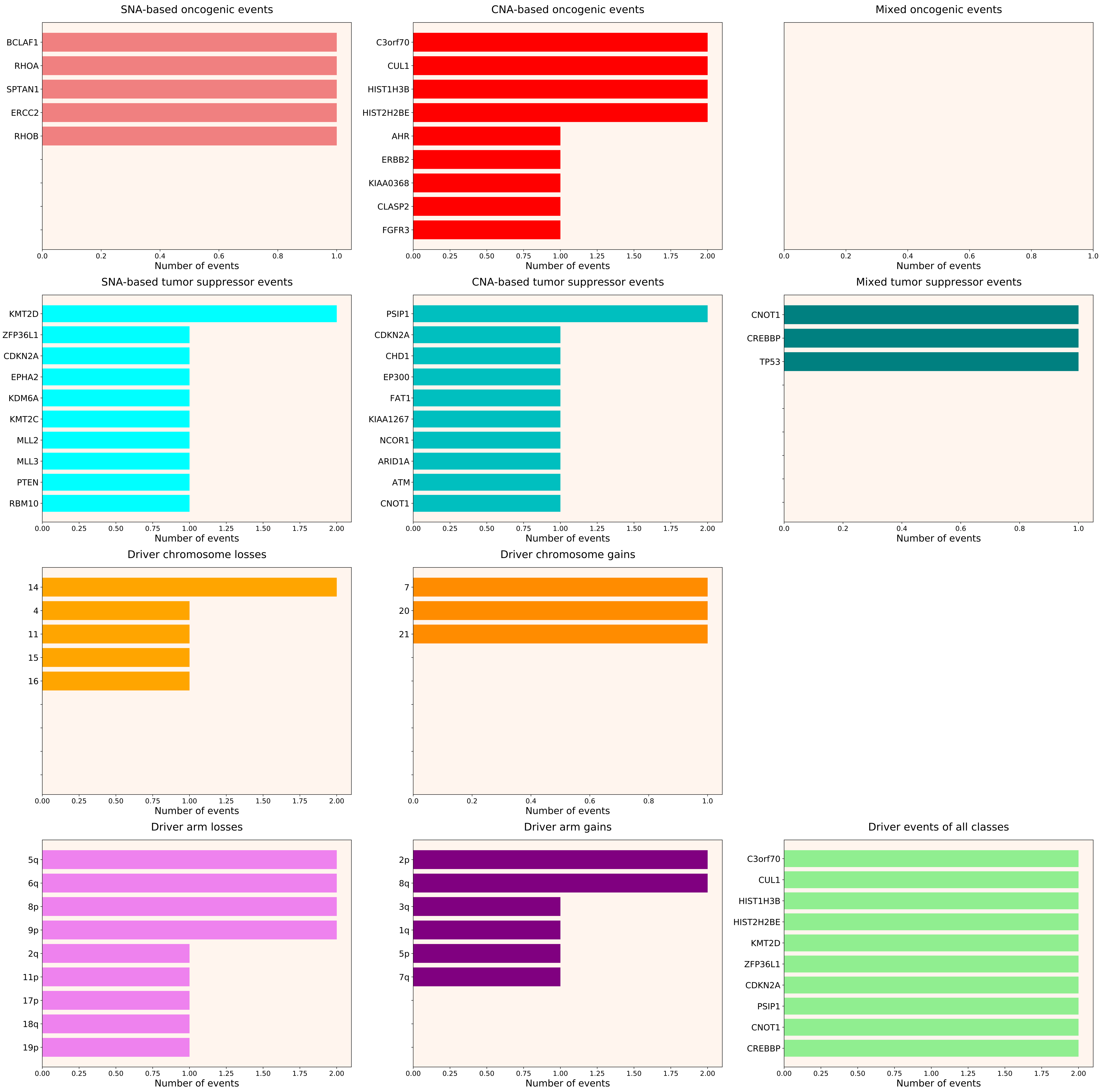
SNA-based oncogenic events
BCLAF1
RHOA
SPTAN1
ERCC2
RHOB
0.0
0.2
0.4
0.6
0.8
1.0
Number of events
CNA-based oncogenic events
C3orf70
CUL1
HIST1H3B
HIST2H2BE
AHR
ERBB2
KIAA0368
CLASP2
FGFR3
0.00
0.25
0.50
0.75
1.00
1.25
1.50
1.75
2.00
Number of events
Mixed oncogenic events
0.0
0.2
0.4
0.6
0.8
1.0
Number of events
SNA-based tumor suppressor events
KMT2D
ZFP36L1
CDKN2A
EPHA2
KDM6A
KMT2C
MLL2
MLL3
PTEN
RBM10
0.00
0.25
0.50
0.75
1.00
1.25
1.50
1.75
2.00
Number of events
CNA-based tumor suppressor events
PSIP1
CDKN2A
CHD1
EP300
FAT1
KIAA1267
NCOR1
ARID1A
ATM
CNOT1
0.00
0.25
0.50
0.75
1.00
1.25
1.50
1.75
2.00
Number of events
Mixed tumor suppressor events
CNOT1
CREBBP
TP53
0.0
0.2
0.4
0.6
0.8
1.0
Number of events
Driver chromosome losses
14
4
11
15
16
0.00
0.25
0.50
0.75
1.00
1.25
1.50
1.75
2.00
Number of events
Driver chromosome gains
7
20
21
0.0
0.2
0.4
0.6
0.8
1.0
Number of events
Driver arm losses
5q
6q
8p
9p
2q
11p
17p
18q
19p
0.00
0.25
0.50
0.75
1.00
1.25
1.50
1.75
2.00
Number of events
Driver arm gains
2p
8q
3q
1q
5p
7q
0.00
0.25
0.50
0.75
1.00
1.25
1.50
1.75
2.00
Number of events
Driver events of all classes
C3orf70
CUL1
HIST1H3B
HIST2H2BE
KMT2D
ZFP36L1
CDKN2A
PSIP1
CNOT1
CREBBP
0.00
0.25
0.50
0.75
1.00
1.25
1.50
1.75
2.00
Number of events
